# Rupture of the Pregnant Uterus

**Published:** 1872-05-15

**Authors:** 


### Original Translations.

### RUPTURE OF THE PREGNANT UTERUS.

TRANSLATED FROM SCHMIDT'S "JAHRLUEHER," BY DR. MARY J. SAFFORD.

A woman in the 7th month of her 16th pregnancy, from lifting a heavy weight was suddenly seized with a severe pain in the hypogastric region, and with vomiting, which prostrated her exceedingly in a few hours.

The same symptoms, except aggravated, occurred the second day; and now the diagnosis of acute peritonitis was made; and by blood-letting the symptoms were somewhat ameliorated.

The 3d day vomiting returned, with typhoid appearances; the tongue dry and dark; skin hot; pulse increased; with great prostration.

The abdomen was much distended, and especially prominent in the region of the navel.

In the left epigastric, umbilical, and hypogastric regions, was to be felt a somewhat elastic tumor, that gave a dull sound upon percussion.

Tense membranes were felt in the os.

Nowhere were the child's extremities to be felt. Neither the heart tones to be heard.

From symptoms and appearances there was no doubt that the parenchyma of the uterus was ruptured, with peritoneal covering, in consequence of which acute peritonitis had occurred.

That the death of the fœtus had been accompanied by a rapid decay that caused the decided accumulation of gas within the membranes, and also led to septæmia.

The membranes were punctured, when a large quantity of offensive-smelling gas escaped, accompanied by a not less offensive brownish yellow fluid. This was followed by the decayed fœtus.

After this operation, the patient was very comfortable, and in six weeks was quite convalescent.

EXTRA-UTERINE PREGNANCY.

A woman aged 34, after a pause of 14 years, became pregnant again. In the 7th month, she was seized with excruciating pain in the hypogastric region, seeming more severe in the fundal region of the uterus, where one thought to feel the fœtal extremities.

The patient complained of a peculiar sensation, as if the child turned itself from left to right.

The fœtal heart-tones were to be heard more distinctly than usual in the right iliac region.

Nothing was to be felt by a vaginal examination.

The physician was called, and found the patient insensible. She had fallen in a fainting-fit, from which she soon died.

An autopsy revealed the child in the peritoneal cavity, an apparent fœtus at full term, lying in a considerable quantity of fluid and of coagulated blood.

In following the navel-string one came, to the left, upon a ruptured cyst, which presented the exact appearance of a pregnant uterus. This was imbedded between the layers of the broad ligaments. Its walls were richly supplied by blood-vessels from the uterus.

The placenta was fastened to the side of the cyst.

The left fallopian tube was enormously enlarged, ran over, and ended with some of the fimbrice internal to the cyst.

A left ovary was not to be found. The uterus was decidedly enlarged, its mucous membrane thickened and softened, and presented a half disorganized appearance, that bespoke the existence of a decidua.

HOSPITAL GANGRENE AND ITS TREATMENT WITH CAMPHOR-GUM.

A. Netter, in Riemes, is of the opinion that hospital gangrene is a peculiar destruction of the subcutaneous and intermuscular, cellular and fat tissues.

The destroyed mass contains, therefore, much fat. The camphor unites with this at a low temperature, and forms a fluid substance —a camphor oil. This is discharged; and then comes to view the tissue capable of life.

Netter says if there are no anatomical lesions, aponeurosis, fascial and otherwise, neither inflammation, complicated with erysipelas, nor pyæmia, that camphor, early applied and in large quantities, is a sure cure for hospital gangrene. He cites a large number of cases successfully treated in this way. Eight cases that were cured are fully described.

He also tried, with equally good results, camphor powder upon phagedenic chancres that had assumed a gangrenous form.

From his remarks we infer that the spread of hospital gangrene rests upon a ferment, whose life camphor powder seems to cut short.

He tried in two cases the application of quinine and charcoal, but without the desired result.

He considers the disease a purely local one, and that the general *malaise* accompanying it, is only in consequence of local disease, the re-absorption of putrid material. He considers the application of ferr. sesq. chlor. as dangerous, and recommends camphor-gum as the most effective remedy in his hands yet tried.

A MONUMENT to the memory of Professor Schuh is to be erected in Vienna. Four thousand four hundred and five florins have already been raised toward it.

THE mortality of Vienna the second week in April was much greater than usual. There were 562 deaths, 80.1 per cent. One hundred and fifty-eight died of tuberculosis. There were 9.7 cases of small-pox daily. There was a typhus epidemic, not an unusual thing here, but more severe and fatal than common. Only two cases of spotted fever have been observed. They were in Rudolph's hospital.

INTRA-UTERINE VARIOLA.—In the *Soc. des Sciences Med.*, of Lyons, M. Poucet showed recently a fœtus aborted by a woman attacked by varioloid. This fœtus presented a disseminated pustular eruption over different parts of the body. Dr. Molliere mentioned that two women had variola during the three first months of their pregnancy. These children were born at full term. He vaccinated both of them on the eighth day, with some other children, but in neither of them did the vaccine take. In one of these cases the variola was confluent, in the other very slight.—*The Doctor.*

